# The Game for Three: *Salmonella*–Host–Microbiota Interaction Models

**DOI:** 10.3389/fmicb.2022.854112

**Published:** 2022-04-18

**Authors:** Krzysztof Grzymajlo

**Affiliations:** Department of Biochemistry and Molecular Biology, Faculty of Veterinary Medicine, Wrocław University of Environmental and Life Sciences, Wrocław, Poland

**Keywords:** *Salmonella*, microbiome, infection models, host-pathogen interaction, pathogen-microbiota interactions, organoids, mice infection

## Abstract

Colonization of the gastrointestinal (GI) tract by enteric pathogens occurs in a context strongly determined by host-specific gut microbiota, which can significantly affect the outcome of infection. The complex gameplay between the trillions of microbes that inhabit the GI tract, the host, and the infecting pathogen defines a specific triangle of interaction; therefore, a complete model of infection should consider all of these elements. Many different infection models have been developed to explain the complexity of these interactions. This review sheds light on current knowledge, along with the strengths and limitations of *in vitro* and *in vivo* models utilized in the study of *Salmonella*–host–microbiome interactions. These models range from the simplest experiment simulating environmental conditions using dedicated growth media through *in vitro* interaction with cell lines and 3-D organoid structure, and sophisticated “gut on a chip” systems, ending in various animal models. Finally, the challenges facing this field of research and the important future directions are outlined.

## Key Messages

• The GI tract is occupied by trillions of microbes; therefore, a complete model of infection should include interactions between the host, its microbiota, and the infecting pathogen, which defines a specific triangle of interaction.
• Most of the currently used infection models may be applied to investigate the host–*Salmonella*–microbiota triangle.
• Appropriate model selection may shed new light on *Salmonella* infection.

## Introduction

*Salmonella* is a Gram-negative foodborne pathogen with the ability to infect a wide range of species. Depending on the serovar and infected host, *Salmonella* can cause diseases with different clinical symptoms, ranging from alimentary tract disturbances (gastroenteritis) caused by host-unrestricted serovars such as Enteritidis or Typhimurium, to invasive typhoid-like diseases caused by host-restricted serovars, such as Typhi or Gallinarum (Uzzau et al., 2000). To establish successful infection, enteric pathogens, such as *Salmonella*, must sense and respond to newly encountered host environments to regulate the expression of critical virulence factors, such as flagella, fimbriae, invasins, and secretion systems present in *Salmonella* Pathogenicity Islands (SPI) to actively reach, attach, and invade host cells (Saini et al., 2009; de Masi et al., 2017; Kolenda et al., 2019). Interestingly, allelic variations of those virulence factors are often directly related to the phenomena of host specificity (Grzymajło et al., 2013; Yue et al., 2015; de Masi et al., 2017). After adhering to the host cell surface, *Salmonella* actively invades and survives intracellularly in phagocytic and non-phagocytic cells (Malik-Kale et al., 2011). Most studies on the early stages of *Salmonella* infection have focused on a direct interaction with host cells, such as enterocytes, M-cells, or macrophages, in many cases with the use of immortalized cell line models (Brosnahan and Brown, 2012; Torraca et al., 2014; Kolenda et al., 2019). However, colonization of the GI tract by enteric pathogens always occurs in a broader context, strongly determined by host-specific gut microbiota, which can heavily affect host–pathogen interactions. The gut microbiome appears to be one of the critical factors in resistance to enteric pathogen colonization; however, the exact mechanism has not been fully elucidated (Velazquez et al., 2019; Rogers et al., 2021).

**FIGURE 1:**
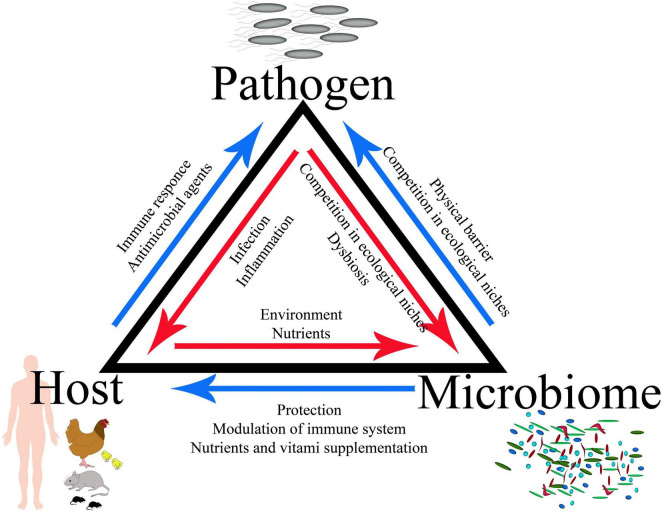
Concept of the host–pathogen–microbiota triangle.

**FIGURE 2:**
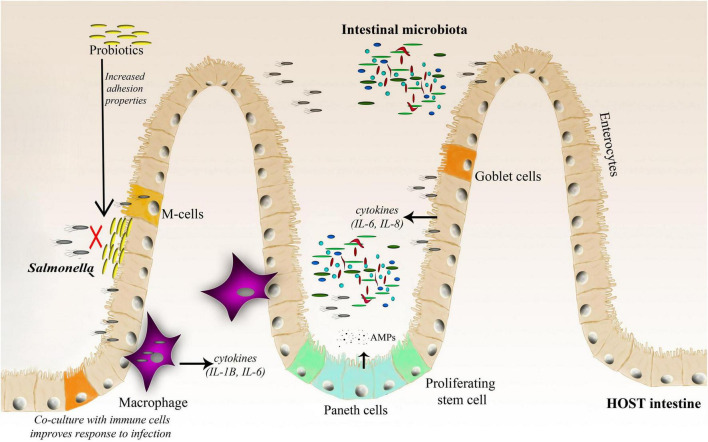
A schematic representation of the *Salmonella*–host–microbiota interaction. This figure highlights various factors (immune response, microbiota adhesion, and microbiota competition with the invading pathogen) affecting the gut microbiota composition and infection outcome.

The GI tract is occupied by trillions of microbes, including complicated bacterial, fungal, and viral populations; therefore, a complete infection model should include the interactions between the host, its microbiota, and the infecting pathogen, which defines a specific triangle of interaction (Figures 1, 2). Bacteria account for around 93% of the unique gene repertoire of the microbiome, viruses less than 6%, and fungi around 0.1% (Shkoporov and Hill, 2019). Despite recent research demonstrating the important role of viral and fungal microbiota (Lecuit and Eloit, 2017; Pérez, 2021; Thielemann et al., 2022), this review will focus on the largest part of the microbiome—bacteria.

*Bacteroidetes* and *Firmicutes* dominate the human gut, accounting for up to 90% of the bacteria in the adult human gut (Jandhyala et al., 2015), however, their composition changes throughout the life. Among the trillions of microorganisms that inhabit the human intestine, there are more than 1,000 species, at least 160 bacterial species per person, and 100 times more genes than in the human genome (Turnbaugh et al., 2007; Gahan et al., 2011). The complexity and diversity of the microbiome are determined by multiple factors, such as age, genetics, diet, pharmacological treatments, and general lifestyle (Vasquez et al., 2018; Zhang et al., 2018). There are two major features of microbial communities that are postulated to mediate resistance to pathogens: community species richness and community composition. A healthy and balanced intestinal microbiome provides many benefits to the host, such as proper development of the immune system, stimulation of proper intestinal tract cell development (Salzman et al., 2010), absorption of nutrients, support for vitamin production, and finally protection against pathogenic infections (Huttenhower et al., 2012). Disruption of the microbiota (known as dysbiosis) due to various factors, such as antibiotic treatment or an unbalanced diet, favors infection by different pathogens, including *Salmonella.* Gut microbes promote colonization resistance by competing with pathogens for nutrients, priming, modulating the host immune system, and directly targeting other microbes with metabolites (Vasquez et al., 2018). Furthermore, the microbiota can act as a physical barrier against invading bacteria by blocking pathogen access to the epithelial layer (Sassone-Corsi and Raffatellu, 2015), and actively stimulates Paneth cells for the production of antimicrobial peptides (AMPs) (Salzman et al., 2007; Yokoi et al., 2019; Figure 2).

Despite numerous studies, there are still many unknowns about the effect of perturbations in the intestinal microbiota on host susceptibility to invading pathogens. Enteric pathogens, such as *Salmonella*, can interact intensively with the intestinal microbiota, thereby affecting the composition of microbiome, which changes the outcome of the infection (Sekirov et al., 2008; Bucci et al., 2016). Several unanswered questions have arisen over the years about *Salmonella*–host–microbiome interactions (Table 1):

**TABLE 1:** Lesson learned from different models for *Salmonella*-host-microbiota interactions.

	Biological questions for Host-*Salmonella*-microbiota								
	
Experimental model	Q1	Q2	Q3	Q4	Q5	Q6	Q7	Q8	Q9
Growth media									
Anaerobic culture systems:									
Batch (bacterial coctail)									
Continous flow									
Agar overlay									
Two-compartment systems									
Cell lines:									
Simple interaction									
Transwell									
3D culture models									
Organoids									
IVOC									
Organ on a chip									
Animal models:									
Mice									
Chicks									
Pigs									

*Rows represent the experimental models for Salmonella -host-microbiota interaction described in this review. Columns represent the following biological questions that can or cannot be answered by those methods. Color code: green—best in the category; red—worst in the category; yellow—moderate in the category; gray—not applicable).*

*(Q1) What are the proper media and growth conditions for Salmonella—microbiome interactions?*

*(Q2) How and when does Salmonella utilize its virulence factors in the contact with the microbiome?*

*(Q3) How do Salmonella and microbiota compete for nutrients, and environmental niches?*

*(Q4) How the microbiome protects the host from Salmonella?*

*(Q5) How does Salmonella infection impact the composition of the intestinal microbiota and infection outcome?*

*(Q6) What is the role of the microbiota composition and richness in colonization resistance and host protection against Salmonella?*

*(Q7) How does the microbiome (defined as microbiota and its environment) affect/interfere with the host’s innate immune response during Salmonella infection?*

*(Q8) How Salmonella can target the host to manipulate the environment inhabited by microbiota?*

*(Q9) How Salmonella infection can be used as a model system for dysbiosis?*

Q1) What are the proper media and growth conditions for *Salmonella*–microbiome interactions?

Q2) How and when does *Salmonella* use its virulence factors in contact with the microbiome?

Q3) How do *Salmonella* and microbiota compete for nutrients and environmental niches?

Q4) How does the microbiome protect the host from *Salmonella*?

Q5) How does *Salmonella* infection impact the composition of the intestinal microbiota and the outcome of infection?

Q6) What is the role of the microbiota composition and richness in colonization resistance and host protection against *Salmonella*?

Q7) How does the microbiome (defined as microbiota and its environment) affect/interfere with the host innate immune response during *Salmonella* infection?

Q8) How can *Salmonella* target the host to manipulate the environment inhabited by the microbiota?

Q9) How can *Salmonella* infection be used as a model system for dysbiosis?

Many different infection models have been developed to address these questions and to understand this three-way gameplay between the pathogen, host, and microbiota. This review addresses the current knowledge, along with the strengths and limitations of *in vitro* and *in vivo* models used in the study of *Salmonella*–host–microbiome interactions.

## Models for Host–Pathogen–Microbiome Research in *Salmonella* Studies

**FIGURE 3:**
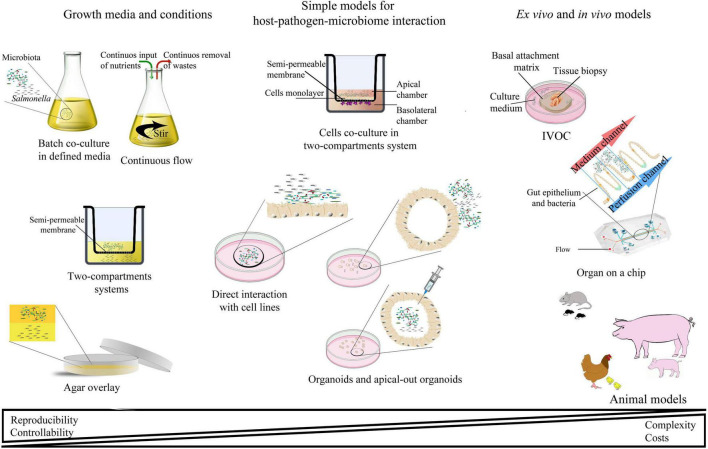
Schematic representation of the most abundant interaction models for studying host–pathogen–microbiota interactions.

Many experimental models have been developed to study *Salmonella* infections, ranging from *in vitro*, *ex vivo*, and finally *in vivo* studies (Table 2 and Figure 3). Most of them have been successfully applied to study *Salmonella* interactions with the microbiota or the host–*Salmonella*–microbiota triangle. *In vitro* experiments focusing on environmental cues and stimuli that mimic the gut environment are less expensive, simpler, and can be a good alternative to more complicated models, especially in the screening phase (Vartoukian et al., 2010). When more complex interaction patterns are considered, established intestinal cell lines can serve as a model for host–pathogen–microbiota interactions during the early stages of bacterial pathogenesis (Brosnahan and Brown, 2012; Carey and Kostrzynska, 2013). Immortalized cell lines can serve as a good and cost-effective model for the early host–microbiota screening experiments; however, due to their relative lack of complexity, the sophisticated architecture of the intestine cannot be fully simulated. To mimic the complex organization of intestinal epithelia, a three-dimensional (3D) organotypic model (Höner zu Bentrup et al., 2006) and an *in vitro* organ culture (IVOC) model (Fang et al., 2013) have been developed to better represent the characteristics associated with intestinal epithelia *in vivo.* In contrast to two-dimensional (2D) cell cultures, the 3D organotypic model has a better organization of junctional, extracellular matrix, brush-border proteins, Paneth cells secreting AMPs, and highly localized mucin production (Höner zu Bentrup et al., 2006; Yin and Zhou, 2018; Gieryńska et al., 2022). In addition, its ability to co-culture with immune cells, such as macrophages, enables the formation of a more physiologically relevant intestinal model, which is more appropriate to study host–bacteria interactions in more detail and the immune responses caused by the microbial, probiotic, and pathogenic infection. On the microbial side, organoids and enteroids have been used for host–pathogen and host–commensal studies (Aoki-Yoshida et al., 2016; Hill and Spence, 2017; Co et al., 2019). *In vitro* experiments are key to understanding the mechanisms of infection in the context of microbiota; however, it is difficult to extrapolate these results to the animal intestinal tract where other factors, such as peristaltic movement and complete host defense system, could interfere with the process. Moreover, it is not an easy task to transplant the complex microbiota communities living in the gut to a relevant *in vitro* system, mostly because of its limited viability and high sensitivity to oxygen. Therefore, animal models are frequently used to explore the course and mechanisms of *Salmonella* infections in the context of their interactions with the microbiota.

**TABLE 2:** Comparison of various characteristics between experimental models.

Experimental model	Cost	Ethical issues	Controllability	Reproducibility	Capacity for real-time monitoring of gene/protein expression	Reproduction of physiological conditions	Capacity for long term investigation	Microbiota diversity	Cellular diversity (host cells)	Capacity for genetic manipulations (host cells)	Spatial complexity	Spatial heterogeneity	Personalized treatment
Growth media													
Anaerobic culture systems:													
Batch (bacterial coctail)													
Continous flow													
Agar overlay													
Two-compartment systems													
Cell lines:													
Simple interaction													
Transwell													
3D culture models													
Organoids													
IVOC													
Organ on a chip													
Animal models:													
Mice													
Chicks													
Pigs													

*Rows represent the experimental models for Salmonella -host-microbiota interaction described in this review. Columns represent an experimental feature that can be addressed with the use of the model. Color code: green—best in the category; red—worst in the category; yellow—moderate in the category; gray—not applicable.*

In deciding which model should be applied to answer experimental questions, one needs to find a balance between simple systems, which paint a limited picture of the interaction but are easy-to-maintain and control, and highly complex models that fulfill many different aspects of the interaction, but are more difficult to control and utilize (Table 2 and Figure 3). The incorporation of a third player, a complex microbiome population, generates additional limitations and challenges; however, it leads to the generation of a big picture of pathogenic infection.

## Growth Media and Anaerobic Culture Systems

*In vitro* studies that simulate the gut environment are useful for investigating the mechanisms of pathogen–microbiota interactions by mimicking the host milieu. These experiments are less expensive, simpler, provide better control, enable easier manipulation, have the potential to find appropriate conditions to analyze specific bacterial interactions, and, finally, require no ethical restrictions (Table 1). However, such *in vitro* experiments also have disadvantages. For example, among the microbiome, many bacterial species are considered uncultivable (Eckburg et al., 2005; Vartoukian et al., 2010). This last statement is only partially true as most microorganisms are amenable to cultivation under appropriate culture conditions. Some anaerobes can survive in oxygen-rich conditions by forming spores (Kato et al., 1997). Others, such as many gut-inhibiting species, are obligate anaerobes and can be cultured only in oxygen-free conditions (Vartoukian et al., 2010).

Even the selection of growth media suitable for complex microbiota cannot ensure that these conditions are suitable for enteric pathogens, such as *Salmonella*. For example, selenite-rich media enhance the growth of *Salmonella* species but inhibit fecal streptococci (Ito et al., 2019). Growth media need to be modified to obtain an appropriate concentration of proteins, sugars, and/or salts to mimic conditions in the ileum or colon. Many other features, such as pH, temperature, oxygen level, and different energy sources, affect media selectivity. Correctly selected media allow the growth and detection of less abundant bacteria, which may be missed in culture-independent studies. However, despite experiments with different culture media and conditions, microbiota composition from fecal inoculum differs in growth reactor cultures due to changes in the environment, such as medium composition, pH, or retention time (Cleusix et al., 2008; Ma et al., 2010; Naimi et al., 2020).

There are several strategies to provide appropriate media and growth conditions for microbiota-*Salmonella* interactions. The easiest solution is to measure how the selected environment affects both sides of the interaction: microbiota composition and richness, and the *Salmonella* proliferation and gene expression profile. Frequently, bacteria recovered from fecal samples are diluted in phosphate buffer and inoculated with selected media. Initially, commercial non-selective media, such as 5% sheep blood agar, M9, gut microbiota medium (GMM), and gut anaerobic medium (GAM), were used. The microbiota were grown under a range of oxygen availability (aerobic conditions, aerobic conditions with 2.5% CO_2_ or 5% CO_2_, microaerophilic conditions, and anaerobic conditions) at different temperatures and with different incubation times (from hours to months) (Rettedal et al., 2014). Subsequently, specific media were suggested for *in vitro* interactions between *Salmonella* and the microbiota. For example, *Saccharomyces cerevisiae* fermentation product (*SCFP*) supplemented with XPC (*Original XPC™ Diamond V, Cedar Rapids, IA, United States*) significantly reduced the population of *Salmonella* Typhimurium, suggesting that XPC enhances the inhibition of *Salmonella* growth by the chicken cecal microbiota (Roto et al., 2015; Park et al., 2017).

With the exception of dedicated media, there is still a need for systems that can closely mimic the *in vivo* situation, and therefore reproduce the physiological parameters of the GI environment. To date, several *in vitro* culture methods have been applied to study the interaction between the intestinal microbiota and invading pathogens, including *Salmonella.* The simplest model is described as a “bacterial cocktail” in which defined GI microbiota and invading pathogens are mixed in one compartment. Batch cultures that mimic the conditions of the intestine are frequently used as models of the impact of microbiota on *Salmonella* growth. For example, batch inoculation of fresh fecal bacteria with *S*. Typhimurium severely affects pathogen survival (Levine et al., 2012; Avendaño-Pérez et al., 2015). Similar systems were used to investigate the capacity to inhibit *S.* Typhimurium growth by 973 anaerobic bacterial culture supernatants and 16 species isolated from pig feces (Levine et al., 2012). Researchers connected this 1,000 up to 100,000-fold *Salmonella* growth inhibition with the production of fermentation acids during anaerobic growth; therefore, the pH was reduced to 5 or less. Decreased pH caused by probiotic *Lactobacillus* strains as a reason for *Salmonella* growth inhibition was also confirmed in the co-incubation model (Fayol-Messaoudi et al., 2005), co-culture models (Adetoye et al., 2018; Burkholder et al., 2019), and by a modified agar overlay method (Adetoye et al., 2018). In the last one, agar plates incubated under microaerophilic conditions were further covered with *Salmonella* diluted in soft agar [Mueller Hinton (MH) soft agar (0.7% agar)], followed by measuring the size of the growth inhibition zone.

Another frequently used system for pathogen–microbiota interaction, a continuous flow model, brings substantial advantages to the topic. First, it simulates transit in the intestine, and second, it supports fresh substrate supplementation and the removal of toxic products (Ushijima and Seto, 1991; Avendaño-Pérez et al., 2015; Fehlbaum et al., 2015; Poeker et al., 2019). The continuous culture model for *Salmonella*–microbiota interaction was based on the original Macfarlane model (Macfarlane et al., 1998) inoculated with diluted feces. Despite its limited microbial stability, this system was used in experiments with enteropathogens. Initially, exogenous bacteria were washed out of the system (Carman and Woodburn, 2001; Payne et al., 2003). Modification of this model, with fecal microbiota immobilized on gel beads in continuous-flow anaerobic cultures (Cinquin et al., 2006a,b) was successfully applied for further investigations with *Salmonella* (Le Blay et al., 2009; Dostal et al., 2014). Researchers suggest that the immobilization of *Salmonella* on polysaccharide beads allows maintenance of pathogens even for long incubation periods, up to 43 days. Another proposal was to immobilize fecal microbiota instead of pathogens to prevent instability of the microbiome diversity. These Poly-FermS results with high stability and reproducibility were used for microbiota–pathogen interactions (Zihler Berner et al., 2013), including studies on *Salmonella*–microbiota interactions (Fang et al., 2013).

In addition to direct cell–cell interactions, the impact of cell-free microbiota supernatant on *Salmonella* growth and proliferation can be measured. Its antibacterial activity is determined either by direct incubation with the membrane filtration-sterilized supernatant or by using two-compartment systems. In the former case, *Lactobacillus plantarum* supernatant added to selected foodborne pathogens, including *Salmonella* Paratyphi A and *S*. Typhimurium SA2093 in serial dilutions, was shown to result in anti-*Salmonella* activity (Uraipan et al., 2014). Other studies have shown that heat-inactivated cell-free supernatant (CFS) of selected probiotics reduces the cytotoxicity of *S.* Typhimurium and inhibits its growth by approximately 50%. Moreover, the expression of the *IL-8* gene initially induced by *Salmonella* was also reduced (Kawarizadeh et al., 2020).

The second method involves two-compartment systems, in which two bacterial populations are separated by a permeable or semi-permeable membrane and share only the growth medium. A high density of *Escherichia coli* was shown to inhibit *S*. Typhimurium growth in such settings (Avendaño-Pérez et al., 2015). In addition, the high-density population of *Salmonella* may inhibit microbiota growth, as *S*. Typhimurium inhibits *Lactobacillus gasseri* and *Bifidobacterium bifidum* growth in the other compartment (Avendaño-Pérez et al., 2015). On the other hand, using the comparison between batch and the two-compartment systems, it was shown that the growth inhibition of *S.* Typhimurium depends on direct cell–cell contact rather than responding to metabolites released in the medium (Avendaño-Pérez and Pin, 2013). This study demonstrates the existence of a novel interaction scheme between the intestinal microbiota and *S.* Typhimurium, which requires cell contact or proximity and leads to growth inhibition or loss of the cultivability of *S.* Typhimurium. This phenomenon may be related to the activity of the Type 6 Secretion System (T6SS), which is used by GI pathogens to compete with the intestinal microbiota (Sana et al., 2016; Anderson et al., 2017; Wood et al., 2020).

Collectively, the selection of appropriate growth media and growth conditions allows an understanding of the mutual interactions between the pathogen and the microbiota at the basic level, including competition for nutrients, metabolites released by the microbiome, and virulence factors expressed by *Salmonella* during contact with other bacteria.

## Cell Lines

Several studies have shown that probiotics and microbiota species can affect *Salmonella* growth and gene expressi*on in vitro*. Established cell lines are well-tested and relatively easy-to-maintain models for the introduction of host cells into the *Salmonella*–microbiota equation. Moreover, the relative simplicity of cell line models allows investigation of the molecular mechanisms of microbiota activity in this interaction. Immortalized cell lines can serve as a good and cost-effective model for early screening experiments; however, due to their lack of complexity, they cannot fully mimic the complex architecture of the intestine. The major goals of such studies are to explain the immunomodulatory effect of various probiotic strains, to investigate the expression of virulence factors in *Salmonella* in the presence of microbiota, and, finally, to select strains that can effectively affect *Salmonella* adhesion to and invasion of host cells. These experiments were mainly conducted using intestinal epithelial cells (IECs), including the cell lines of both normal tissue origin and cancerous origin from different species (see below).

The simplest model used in *Salmonella*–host–microbiota interactions is confluent cell monolayers of IECs, such as IPEC-J2 (Schierack et al., 2011), IEC-6, or IEC-8 (Khailova et al., 2010; Liu et al., 2010) or cells of cancerous origin, such as HT-29 and Caco-2 (Carey and Kostrzynska, 2013; Kawarizadeh et al., 2021). Cell lines of normal tissue origin are morphologically and functionally similar to primary cells, develop proper microvilli, tight junctions, and Toll-like receptors (TLRs). Moreover, its innate immune response is relatively closer to primary tissue and therefore better mimics the host physiology (Schierack et al., 2006; Brosnahan and Brown, 2012). Some cell lines, like HT29-MTX cells, have the ability to differentiate into goblet cells and secrete mucin (Lesuffleur et al., 1993; Leteurtre et al., 2004). It is worth mentioning that complete confluence of cells plays an important role in such experiments because the empty spaces between the cells provide additional niches for bacteria. On the other side, well-developed tight junctions between confluent cells may act as entry sites for *Salmonella* (Fattinger et al., 2020).

One of the useful approaches of the cell line model is to measure the release of pro- and anti-inflammatory molecules in response to *Salmonella* infection and to explain the immunomodulatory effects of various microbiota. The expression profile can be measured either based on the mRNA level *via* quantitative polymerase chain reaction (qPCR) or at the protein level based on released cytokines (Carey and Kostrzynska, 2013; Moshiri et al., 2017; Kanmani and Kim, 2020). Probiotic strains (microbiota) added to confluent cell monolayers before, during, or after *Salmonella* infection for the defined period modulate cytokine expression profiles in the infected/growth media. For example, prestimulation of the HT-29 cell line monolayer with *Lactobacillus brevis*, *Lactobacillus curvatus*, and *Lactobacillus pentosus* for 48 h, followed by washing steps and infection with *S*. Typhimurium for either 3 or 12 h, there was a general reduction of the inflammatory response (Kanmani and Kim, 2020). It is worth mentioning that the order of infection plays a role in host–*Salmonella*–microbiota interactions. Preincubation, co-incubation, and postincubation of probiotic strains may affect *Salmonella* infection in a different way. For example, only preincubation of probiotic *Lactobacillus* and *Bifidobacterium* strains with the HT-29 cell line followed by *S*. Typhimurium D104 infection had an immunosuppressive effect on IL-8 mRNA expression and IL-8 secretion (Carey and Kostrzynska, 2013). Furthermore, in the case of *Lactobacillus kefir* IM002, the anti-inflammatory effect was caused by incubation with the bacterial supernatant. The probiotic strain and its supernatant were also tested by Bermudez-Brito et al. (2013) *Bifidobacterium breve* co-incubated with *Salmonella* Typhi and dendritic cells (DCs) monolayers significantly decreased the secretion of pro-inflammatory cytokines, suggesting that whole bacteria promote anti-inflammatory effects and prevent *Salmonella*-induced inflammation, whereas the secreted components (CFS) exert anti-inflammatory effects in the GI tract. Kanmani and Kim (2020) revealed that in the case of *Lacticaseibacillus paracasei* CNCM I-4034 and DC cell, probiotic bacteria as well as CFS, activates TLR signaling and decreases the production of proinflammatory cytokines in response to *S*. Typhi infection.

In addition to the host cell response, the expression of *Salmonella* virulence genes can be measured during cell line infection in the presence of probiotic strains. *Lactobacillus acidophilus, Lactobacillus rhamnosus*, and *Lactobacillus casei* were shown to affect the expression of *S*. Javiana virulence genes *invA*, *prgH*, *pltA*, and *cdtB* in the presence of HT29-MTX cells at the mRNA level (Burkholder et al., 2019). Moreover, the abovementioned probiotics reduce *S*. Javiana invasion and limit *Salmonella*-induced cell damage (Burkholder et al., 2019).

Adhesion to host cells, followed by invasion, is one of the crucial steps in *Salmonella* pathogenicity. Attachment to host proteins and gut mucosa can be affected by various types of gut microbiota, which compete for binding sites present on host proteins (Das et al., 2013; Uraipan et al., 2014). For example, the *L. plantarum* KSBT 56 strain isolated from dahi chenna (a fermented milk product) co-administered or 1 h after the pathogen prevents not only *Salmonella* Enteritidis adhesion to the HCT-116 colon epithelial cell line, but decreases its biofilm formation ability (Das et al., 2013). Preincubation with *L. plantarum* CIF17AN2 isolated from healthy infant feces decreased the binding of *S.* Typhimurium SA2093 to HT-29 cells (Uraipan et al., 2014). In this particular case, *Lactobacillus* strains applied before *Salmonella* work more efficiently, and therefore suggest that prebiotic adhesion may block *Salmonella* entry sites. *Bacillus coagulans* also significantly reduced the binding of *S*. Typhimurium to HT-29 cells, and no effect was observed for the supernatant at different concentrations (Kawarizadeh et al., 2019). Interestingly, the addition of 1–4% freshly prepared fecal slurry under anaerobic conditions limited this anti-*Salmonella* activity, whereas supplementation with saba starch significantly decreased the number of attached *Salmonella*. This phenomenon was caused by a lower production of fatty acids and therefore a decrease in pH. Lower adhesion levels may thus be connected either with the direct competition of bacteria for binding spots or by chemicals released by the microbiota.

The idea that probiotics block *Salmonella* binding sites was confirmed using IPEC-J2, a porcine IEC line isolated from neonatal piglet mid-jejunum, widely used as a model of host–pathogen interaction and supporting the invasion of at least several *Salmonella* serovars (Schierack et al., 2006; Brosnahan and Brown, 2012; Klasa et al., 2020). Moreover, due to the expression of some pathogen recognition receptors reported (Crocker and Feizit, 1996; Skjolaas et al., 2007), IPEC-J2 is a good model for competition between microbiota and *Salmonella* for binding sites. For example, preincubation and co-incubation with *E. coli Nissle* 1917 (EcN) and *Salmonella* resulted in suppression of invasion by decreased adhesion (Schierack et al., 2011). In addition to adhesion and invasion, the presence of the microbiota in host–*Salmonella* interactions affects the cytotoxicity caused by pathogens in a simple co-culture model. The cytotoxicity of *S*. Typhimurium on HT-29 cells with 90% confluency was significantly reduced when incubated with *B. coagulans* in comparison to cell-free probiotic supernatant (Kawarizadeh et al., 2019).

To further investigate the impact of microbiota growth media/supernatant on *Salmonella*–microbiota interactions, physical separation of bacteria and cells is required. Among the many systems that separate two compartments, Transwell is frequently used in host–pathogen–microbiota interactions (Tyrer et al., 2011; Bermudez-Brito et al., 2013; Yeung et al., 2013). This system was applied to verify the protection delivered by *Lactobacillus* strains to the Caco-2 cell line stimulated by *S.* Typhimurium lipopolysaccharide (LPS) (Yeung et al., 2013). *Lactobacillus* was found to protect tight junctions and, therefore, epithelial barrier integrity from damage caused by LPS. Another interesting use of the system is the co-culture of two different cell lines in separate compartments (Bermudez-Brito et al., 2013). For example, Caco-2 cells cultured in the upper part and human intestinal-like DCs cultured in the lower chamber mimics the *in vivo* conditions under which DCs open tight junctions between epithelial cells and take up bacteria directly from the intestinal lumen (Rescigno et al., 2001; Fattinger et al., 2020). DCs react differently to probiotics and pathogens in the presence and absence of IECs; therefore, co-culture models such as the one presented above may facilitate the study of host–microbe interactions. Moreover, the transwell system provides a feasible platform for the development of novel probiotics or other agents for further animal studies and clinical trials. However, the transwell system has several disadvantages: the lack of direct cell–cell contact, problems with proper migration due to the size of the pores, or inappropriate differentiation due to weak indirect cell–cell contact (Spoetti et al., 2007; Tyrer et al., 2011). Some of these caveats can be addressed by transwell co-culture models, in which bacteria, immune cells, and epithelial cells are studied together. Finally, cell line models for *Salmonella*–microbiota interactions can help address questions regarding the impact of such interactions on host receptor composition, organization, or modification. For example, alterations in cell glycosylation or fucosylation profiles by *Salmonella* may indirectly affect gut microbiota homeostasis (Hao et al., 2020).

Taken together, the introduction of cell lines as a host representation into three-handed gameplay allows for studying direct interaction between all players. Introduction of the element of the immune system, implementation of niches created by cells tight junctions and microvilli, and well as the playground for pathogen–microbiome competition for nutrients make cell lines a useful model for host–pathogen–microbiome interaction studies.

## 3D Culture Models

An experimental system for host–microbe interactions based on 3D organoid cultures may act as a linkage between traditional 2D cell cultures and *in vivo* systems. Due to the relative simplicity of standard cell line models, many aspects of intestinal infections cannot be replicated *in vitro*. In 2009, Sato et al. (2009) identified essential growth factors for organoid culture and started an era of three-dimensional IEC systems *in vitro*. The advantage of these cultures over classical cell cultures includes better organization of cell junctions and an extracellular matrix and better expression of brush-border proteins. Organoids also possess crypt- and villus-like structures (Wilson et al., 2015) and therefore create a close-to-physiological environment for epithelial cells and microbiota (Mahe et al., 2013), providing a great opportunity for investigations focused on a three-way interaction. Unlike animal models, organoids allow the monitoring of host–microbiota interactions in a controlled environment. Furthermore, it provides the ability to work on human-derived cells and even precisely defined patient cells. Overall, the physiological relevance of the system makes organoids a promising technology for infectious diseases and microbiome studies.

Organoids can be derived from pluripotent stem cells or adult stem cells and can reproduce epithelial tissue *in vitro* (Barker et al., 2010). To support 3D growth, organoids are usually cultured in Matrigel, which provides chemical and mechanical support for intestinal stem cells, and cell proliferation is supported by numerous growth factors (Dutton et al., 2019). Organoids have great potential to be an ideal experimental model, as they are composed of patient-derived primary human epithelial cells, and are also accessible to experimental approaches. However, to date, despite numerous reports on organoids in microbiota investigation (reviewed in Bartfeld, 2016) as well as *Salmonella* studies using organoids (Zhang Y. G. et al., 2014; Yin and Zhou, 2018; Co et al., 2019), no study has used such a model for *Salmonella*–host–microbiota interaction. One possible reason for this is that only a few *in vitro* models allow the co-culture of a variety of bacteria with intestinal cells (reviewed in Elzinga et al., 2019). Nevertheless, the potential of the methods presented below creates the possibility that this model can also be efficiently applied in such experiments.

Previously, co-culture of intestinal organoids with microbiota, such as *Lactobacillus*, was demonstrated to have a positive impact on the barrier function of intestinal cells. *L. rhamnosus* enhances organoid proliferation and differentiation (Shaffiey et al., 2016), whereas *Lactobacillus reuteri* D8 stimulates the growth of intestinal organoids and protects against pro-inflammatory tumor necrosis factor α (TNF-α) (Hou et al., 2020). Microinjection of the non-pathogenic *E. coli* strain ECOR2 resulted in widespread transcriptional response and increased epithelial integrity (Hill et al., 2017). Further, short-chain fatty acids generated by *Akkermansia muciniphila* and *Faecalibacterium prausnitzii* strongly modulate transcription in mouse organoids (Arnold et al., 2016).

Organoids, as well as enteroids and colonoids, are reliable models for studying bacterial pathogenesis (Foulke-Abel et al., 2014; Zhang Y. G. et al., 2014). Many studies have used intestinal organoids to study interactions with enteric pathogens, such as *Salmonella* (Zhang Y. G. et al., 2014; Forbester et al., 2015; Wilson et al., 2015; Co et al., 2019). The complexity of organoid structure allows investigation of how pathogens interact with a variety of host cells and affect their function. For example, measurement of *Salmonella* entry into M-cells, stimulation of Paneth cells degranulation (Farin et al., 2014), or disruption of tight junctions (Zhang Y. G. et al., 2014) can be assessed. Both mice and human intestinal organoids infected with *S*. Typhimurium upregulate pro-inflammatory cytokine expression, such as interleukin-1β (IL-1β), TNF, and interleukin 8 (IL-8) (Zhang Y. G. et al., 2014; Forbester et al., 2015), and activate the nuclear factor kappa B (NF-κB) signaling pathway (Zhang Y. G. et al., 2014). However, Wilson et al. (2015) reported that mouse organoid cultures inhibit *S*. Typhimurium growth even 20 h post-infection by α-defensin activity. Interestingly, the ablation of matrix metalloproteinase 7 (Mmp7) in such organoids allows *S*. Typhimurium replication. The role of Peneth cells and secreted AMPs in the context of *Salmonella* and microbiota interaction was also intensively studied. Dysregulation of Peneth cells’ defensin secretion impacts the microbiome composition (Vaishnava et al., 2008), for example, by altering the balance between Firmicutes and Bacterioidates (Yokoi et al., 2019). At the same time, the absence of mice defensins (cryptdins) increases the host susceptibility to *Salmonella* infection (Salzman et al., 2003). Interestingly, the interaction of *Salmonella* with Peneth cells appears to be dependent on the serovar. It was shown that *S.* Typhimurium is able to enter mouse Paneth cells and induce their autophagy (Bel et al., 2017), whereas *S.* Enteritidis decreases autophagy, *via* the action of AvrA effector (Jiao et al., 2020).

The 3D structure of organoids mimics the organization of *in vivo* tissue, including the organization of both basal and apical sides outside and inside the spheroid, respectively (Figure 3). Most host–pathogen interactions using organoids involve microinjection of the bacterium inside the organoid structure. This allows the pathogen to reach its natural entry site and target apical tight junctions (McCracken et al., 2014; Bartfeld, 2016). Despite the technical problems related to precise microinjection, this method gives a chance to model the luminal antimicrobial response and investigate reactive oxygen species (Wilson et al., 2015, 2017). Another method for studying bacteria–organoid interactions is the dissociation of intestinal enteroids onto permeable transwell supports (VanDussen et al., 2015). This 2D culture method of 3D structures allows independent control of apical and basolateral surfaces and has been successfully used to study a co-culture model of enteroids and macrophages, ultimately enabling a thorough investigation of the innate immune response against enteric pathogens (Noel et al., 2017). The newest idea is to generate reverse polarity organoids, with apical surfaces exposed to the culture media and thus easily accessible for potential interactions (Co et al., 2019, 2021). Such inside-out enteroids open possibilities for studying host–pathogen–microbiota triangle in a more diverse and developed, but still relatively easy to control, environment. Organoids are one of the most promising models for host–pathogen–microbiota interactions (Table 2). The growth of the standardized, validated media and culture methods make this system a good balance between traditional 2D cell culture models and more complicated and less predictable animal models. The undeniable advantage of this system is its ability to investigate diverse human cells during infection by human pathogens.

Another interesting 3D model that may be useful for studying the host–pathogen–microbiome interaction is IVOC (Fang et al., 2013). It includes freshly obtained biopsies maintained in culture media under oxygenation to maintain the exchange of gas and nutrients. The material obtained by biopsy allows the study of the interaction of microbes with human tissue; however, it requires the delivery of fresh tissue from a clinic and generates variability in the samples due to different donors. IVOC allows the studies on the host–microbe interaction under physiologically relevant conditions, with the natural architecture of tissues and fully differentiated intestinal cells. Over the years, the traditional IVOC model (Knutton et al., 1984) was sequentially replaced by the polarized IVOC (El Asmar et al., 2002; Collins et al., 2010) with basal and apical sites available for interactions or by different variants adapted for other purposes (reviewed in Fang et al., 2013). Overall, this model provides a substantial advantage over cell cultures and is suitable for studying the interaction between pathogens and host cells (Haque et al., 2004; Schüller et al., 2009; Fang et al., 2013). What is more, it has also been applied to study probiotic bacteria impact on *Salmonella* infection (Collins et al., 2010).

Taken together, 3D infection models provide a unique opportunity to merge the study of a well-developed and diverse cellular mixture with a complexity close to *in vivo* experiments, with a relatively high controllability. From this perspective, most questions regarding host–pathogen–microbiome gameplay may be addressed using 3D models.

## Organ-On-A-Chip

Most of the abovementioned models allow the co-culture of host intestinal cells and microbes for a few hours up to 1 day, which limits their use in long-term experiments. Some limitations can be avoided by using microfluidic models, in which multiple cell layers may be grown on thin chambers with channels divided by a thin membrane. These systems, classified as “organs-on-a-chip” are generated using soft lithography, a method similar to computer chip manufacturing. Currently, the use of multiple channel shapes, different intestinal cell types, fluid flow, and even vascular channels has become a promising model for *in vitro* studies. In addition, microchannels that support laminar fluid flow can mimic intestinal peristaltic movements, allowing a closer approximation of natural mechanical deformations as well as nutrient delivery and toxin removal. Flow shear stress generated by such models allows the development of a denser actin network, increased mitochondrial activity, metabolic enzyme expression, and gene expression profile at a level closer to that of the *in vivo* intestine (Delon et al., 2019). Moreover, spontaneous formation of villus structures and mucus production by epithelial cells can occur (Sung et al., 2011; Shim et al., 2017; Grassart et al., 2019), thereby providing a platform for creating niches for interactions of microbiota with pathogenic bacteria, including *Salmonella* (Costello et al., 2014). Unlike existing *in vitro* models, microfluid channels form complex interactions between aerobic bacteria, anaerobic bacteria, and host tissues. To apply microfluidic models to patient-specific content, intestinal chips with primary cell lines were introduced (Kasendra et al., 2018). These chips can better reproduce the normal epithelial physiology, allow the use of different intestinal cell types, and finally, allow to mimic the host response to bacterial infection and append the model for many possible applications, including inflammation and infection. The first microfluidic system to co-culture the Caco-2 cell line and *L. rhamnosus* for more than 1 week was presented by Kim et al. (2012) and was later replaced by a more sophisticated gut chip (Kim et al., 2016) and HuMix (human–microbial cross talk) models (Shah et al., 2016; Shin and Kim, 2018). A gut chip, which supports epithelial cells as well as capillary endothelium and immune cells, was successfully applied to the co-culture of Caco-2 cells with *L. rhamnosus* GG and enteroinvasive *E. coli* (Kim et al., 2016), demonstrating for the first time that this system can work in host–pathogen–microbiota interactions.

There are some concerns regarding the limited availability and reproducibility of the system between laboratories, mostly because the system is still in the developing stage. Nevertheless, organ-on-a-chip technology is a powerful tool for host–pathogen–microbiota interaction, and thanks to the introduction of flow that simulates peristaltic movements and allows toxin removal bring them closer to *in vivo* models but with the controllability higher than *in vivo* (Table 2).

## Mice

Although salmonellosis outcomes can differ between species, laboratory mice as an experimental model have proven valuable in the development of human medicine. A remarkable advantage of mouse infection models over *in vitro* and *ex vivo* methods is their ability to allow for proper signaling in intestinal cells, polarized and organized microvilli, enormous heterogeneity of cells affected by a microbe, a complete set of AMPs, mucus production, and a natural environment for the microbiota. Notably, different strains of mice show different levels of susceptibility to *Salmonella* infection (Monack et al., 2004; Lawley et al., 2006), and the host genetic background can heavily influence the outcome of infection (Foster et al., 2021). Most research on *Salmonella* pathogenesis has historically used *Salmonella*-sensitive mice, such as BALB/c or BL-6 strains. These mice have a mutation in the *Slc11a1* gene (also known as *Nramp1*), which makes them susceptible to systemic infection by *Salmonella.* The *Slc11A1* mutation causes a defect in ion transport in phagocytic vesicles, which allows *S.* Typhimurium to survive in macrophages (Blackwell et al., 2001). This model mimics the infection of humans with the host-restricted serovar Typhi that causes typhoid fever; however, new humanized mouse models for *S.* Typhi has also been developed (Libby et al., 2010; Song et al., 2010). For non-typhoidal serovars, *Slc11A1* mice strains possess a significant drawback. Even with a very low infection dose, they are rapidly affected within 10 days. Therefore, to study persistence, some researchers have used 129/SvJ or CBA mice that bear a functional *Slc11A1* allele (Tsolis et al., 2011). *S.* Typhimurium persists in the GI tract for more than 30 days in these mice and has been found to persist in the mesenteric lymph nodes and gallbladder (Monack et al., 2004). Moreover, mouse strains with different *Salmonella* susceptibilities also differ in intestinal microbiome composition (Ferreira et al., 2011). For example, microbes overrepresented in 129S1/SvImJ mice, belonging to the phylum *Bacteroidetes*, are associated with better protection against inflammation but do not affect *Salmonella* colonization levels (Ferreira et al., 2011).

Colonization resistance provided by indigenous commensals is an important barrier to infection by enteric pathogens. Treatment with antibiotics, such as streptomycin, before infection has been shown to reduce the infectious dose of *S*. Typhimurium in mice by 100,000-fold and is therefore commonly used in some mouse models of *Salmonella* infection to circumvent colonization resistance (Barthel et al., 2003; Stecher et al., 2005). These mice have symptoms that are more similar to those of human gastroenteritis; however, in contrast to many other species, they do not exhibit massive luminal fluid secretion. The final effect of such treatments depends on the antibiotic type and dose (Ferreira et al., 2011). For example, microbiome disruption in C57BL/6 mice treated with various doses of streptomycin and vancomycin was more uniform for streptomycin treatment than for vancomycin treatment (Sekirov et al., 2008). Most antibiotic pretreatment studies utilize very high doses of streptomycin (20 mg/mouse) to completely disrupt the microbiota; however, even significantly lower doses, which do not affect the total number of intestinal microbes, make mice more susceptible to *S.* Typhimurium infection (Sekirov et al., 2008). Different antibiotics and different doses were also applied to the 129S1/SvImJ mouse model for persistent *Salmonella* infection (Ferreira et al., 2011). Researchers suggested that the microbiota composition of the 129S1/SvImJ strain is affected differently when treated with metronidazole and streptomycin compared to the C57BL/6 microbiome.

Antibiotic treatment also enables an additional opportunity—microbiota transplantation and therefore opens up new possibilities in *Salmonella*–host–microbiome interactions. To address the following, gnotobiotic mice are used as a mammalian model system where defined microbiomes can be used in a semi-controlled environment. Gnotobiotic mice possess a strictly controlled microbiota level and composition, starting from germ-free mice without microbial species, to mice colonized by a single microbial species, up to a small, precisely defined microbial population (Falk et al., 1998). The higher complexity of microbiota has been shown to increase protection against *Salmonella*-induced inflammation (Stecher et al., 2010). For example, a MET-1 defined microbiota population was generated as protection after antibiotic therapies (Martz et al., 2015). MET-1 did not affect *S*. Typhimurium levels in the colon but was found to decrease systemic colonization of C57BL/6 mice by *Salmonella*. Another example is low-complexity microbiota (LCM) mice, germ-free C57Bl/6 mice with altered Schaedler flora (ASF) (Stecher et al., 2010). In this case, microbiota composition is stable even over long periods, and the gut immune system is closer to normal compared to that of germ-free mice. Both gnotobiotic mice and antibiotic pretreatment models have been frequently used in investigations on the basics of colonization resistance generated by microbiota (Woo et al., 2008; Sekirov et al., 2010; Thiennimitr et al., 2011). Overall, mice lacking microbiota, either due to antibiotic treatment or germ-free, as well as mice with a LCM, with up to 20 species, do not show colonization resistance (Endt et al., 2010; Stecher et al., 2010). As streptomycin-pretreated mice do not develop diarrhea, novel one-day-old C57BL/6 mice have been suggested as a good model for *Salmonella* infection (Zhang K. et al., 2014). Moreover, it could be an interesting model from an interaction with the microbiota point of view. In addition to lacking fully developed intestine as well as M-cells, and possessing enterocytes as the major *Salmonella* entry site, neonatal mice do not possess an established intestinal microbiota even without antibiotic treatment.

In addition to colonization resistance (Endt et al., 2010), the intestinal microbiota plays a role in “pathogen clearance” (i.e., the elimination of the pathogen from intestinal niches after infection). After pathogen infection, even if the host fights out the invader, microbiota richness and diversity are usually strongly affected and must return to their original balance. During this re-growth process, microbiota species fight the pathogen, among others, by stimulating the mucosal immune system (Stecher et al., 2005).

Alterations in the microbiota richness and diversity caused by antibiotic treatment may lead to the generation of a “super-shedder” phenotype, characterized by severe mucosal inflammation, high loads of pathogens in the intestine, high levels of fecal shedding (more than 10^8^/g of feces), and rapid transmission of *Salmonella* to naïve case mates (Lawley et al., 2008). The shedding level of individual mice, at least partially controlled by the microbiota, is a known phenomenon in the spread of infectious diseases (Lawley et al., 2008). Previously, it was shown that after streptomycin treatment of C57BL/6 mice, all animals became super-shedders within 24 h after *S*. Typhimurium infection (Barthel et al., 2003). The persistent infection model induces differentiation between non-super-shedder and super-shedder-specific microbiota, resulting in protection against antibiotic treatment in the latter (Gopinath et al., 2014). Despite the mouse model used in *Salmonella*–microbiota interaction experiments, additional complications arise. Unpredictable variations in microbiota populations not only between different mouse strains but also between isogenic mice supplied by different vendors or even by different time cohorts, strongly affect experimental outcomes (Stecher et al., 2010; Franklin and Ericsson, 2017; Velazquez et al., 2019). Therefore, any experiment regarding the microbiota should consider the aforementioned fluctuations.

## Other Animal Models

In addition to the most abundant mice *in vivo* models, there are at least two animal models frequently used to investigate *Salmonella–*microbiota interactions—chickens and pigs. Chick and laying hens are useful models for *Salmonella* infections as poultry farms are severely affected by salmonellosis, and transmission from chicken eggs to humans is a leading cause of foodborne *Salmonella* outbreaks. However, infection with host-unrestricted serovars, such as *S*. Enteritidis, results in localized intestinal inflammation, without electrolyte efflux or diarrhea (Liu et al., 2018). *Salmonella* infection has been shown to impact chick microbiota composition and richness (Juricova et al., 2013; Liu et al., 2018; Menanteau et al., 2018; Kempf et al., 2020), especially when the first contact with the pathogen occurs in the 1st day after hatching.

Notably, the complexity of chicken microbiota increases significantly from day 1 to days 14–19 of life (Crhanova et al., 2011; Stanley et al., 2013; Liu et al., 2018; Khan and Chousalkar, 2020), and its composition depends solely on environmental factors. After day 1 of hatching, the microbiota complexity is usually very low and consists of approximately five different species (Crhanova et al., 2011). In the following days, microbiome complexity increases, reaching up to approximately 40 different species on day 19 (Crhanova et al., 2011; Stanley et al., 2013). Based on the heterogeneity of intestinal microbiota, infection of chicks with *Salmonella* at different time points after hatching leads to different infection outcomes and may shed a different light on the pathogen–microbiota interaction. Neonatal chicks with limited microbiota richness develop intestinal inflammation between 2 and 4 days post-infection with *S*. Enteritidis (Kogut et al., 2016), which is usually followed by systemic spread (Troxell et al., 2015; Litvak et al., 2019). Infection of young (1–4 days old) chicks with *S*. Enteritidis also contributes to delayed microbiota development and an overall reduction in richness and diversity (Juricova et al., 2013; Mon et al., 2015). Rapidly growing pathogens mainly reduced *Clostridiales*, *Lactobacillales*, and *Bifidobacteriales* just 3 days after infection (Juricova et al., 2013).

An interesting model was used by Barrow et al. (2015) in which the established microbiota from 40-week-old hens was transferred to neonatal chicks. This system was found to significantly reduce the variation in *Salmonella* susceptibility between 1-day-old chicks, and make them less susceptible overall. Another strategy that can be used to improve the resistance of neonatal chicks and their microbiota to wild-type *Salmonella* is the administration of attenuated *Salmonella* strains or probiotics (Azcarate-Peril et al., 2018). The last idea is based on competitive exclusion theory, which states that two closely related species cannot occupy the same ecological niche. It was first described in the chicken model by Rantala and Nurmi (1973), when the administration of attenuated *S.* Typhimurium to 1-day-old chicks competitively excludes the next dose of *S.* Typhimurium administered after 24 h. The positive effect of competitive exclusion in the reduction of *Salmonella* colonization was further confirmed using different *Salmonella* serovars and using many different experimental design (Barrow et al., 1987; Callaway et al., 2008; Crhanova et al., 2011).

Pigs are natural *Salmonella* hosts and, as frequently asymptotic carriers of many *Salmonella* serovars, act as a reservoir for human infection (Pires et al., 2017). Pig and human gastrointestinal tracts are very similar, allowing us to bypass the mice model to avoid colitis in the presence of their natural microbiota (Ahmer and Gunn, 2011; Zhang et al., 2013; Drumo et al., 2016). In addition, pigs infected with *Salmonella* develop an acute phase very early and shed a high concentration of *Salmonella* in the feces (Lynch et al., 2017). As in mice (Lawley et al., 2008) and chicks (Kempf et al., 2020) models, pigs can also develop “low-” and “high-shedder” phenotypes. Interestingly, a “low-” and “high-shedder” phenotypes differ significantly in the abundance of microbiota prior to *Salmonella* infection (Bearson et al., 2013; Argüello et al., 2019); this microbiota diversity may lead to the discovery of specific microbiota species and compositions, which may act as a marker for *Salmonella* susceptibility.

The age of potential hosts plays an important role in the selection of an appropriate pig model for our *Salmonella*–microbiota experiment. Interestingly, microbiome composition is very similar among naturally delivered neonates, and is not strongly affected by variation in host genetics or other environmental factors during the first few days of life (Inman et al., 2010). The complexity of the microbiome increases with age (Inoue et al., 2005), and evolves rapidly during GI tract development, shifting from *Bacteroidetes* to *Firmicutes* with age (Mach et al., 2015). Therefore, it may be interesting to study the impact of *Salmonella* on the unstable vs. stable microbiome in swine (reviewed in Gresse et al., 2017). Piglets can also be used as a model to determine the efficiency of probiotic strains against *Salmonella* infections. Administration of selected *Lactobacillus* strains results in reduced severity and lower pathogen loads after *S*. Typhimurium infection (Casey et al., 2007). However, weaned piglets supplemented with the well-characterized probiotic strain, *Enterococcus faecium*, and infected with *S*. Typhimurium did not have different clinical symptoms from the control pigs (Szabó et al., 2009). Bacterial loads in their feces and internal organs were even higher than those in the control. The composition of intestinal microbiota in piglets infected with *S*. Typhimurium was found to be strongly correlated with the infecting strain virulence (Drumo et al., 2016), and the wild-type strain significantly reduced SCFA-producing bacterial strains, such as *Faecalibacterium*, *Roseburia*, *Butyrivibrio*, and *Clostridium* genera. Of note, similar to the mouse model, *Salmonella* exploits inflammation to compete with the piglet microbiota (Chirullo et al., 2015).

Animal infection models, despite their lower level of controllability, bring substantial advantages to host–pathogen–microbiota investigations. In addition to the natural microbiome environment, appropriate physiological conditions, and a fully developed immune system, they provide a unique opportunity to study a dysbiosis process (Table 2). Most of the questions focused on *Salmonella* infection and its three-handed game with the host and the microbiota can be answered using animal models. Infected organisms possess their own natural microbiome, which can also be modified throughout the experiment. Indeed, this allows to involve all of the elements that may play a role in pathogen infection: (1) interaction of the host cells with the complete microbiome (bacteriome, virome, and mycobiome); (2) complex immune system reaction; (3) all available environmental niches; and (4) natural, appropriate physiological conditions for the microbiome and the pathogen.

On the other hand, there are at least two important drawbacks of animal models: ethical restrictions and low controllability of experimental variables.

## Conclusion and Prospects

The traditional approach for investigating host–enteropathogens interactions is currently faced with a third player—trillions of microbes inhabiting GI tissues. In addition to the examination of pathogen gene/protein expression profiles and host responses, it is important to consider the impact of diverse, complicated bacterial, fungal, and viral populations that live in a specific symbiosis with the host.

A healthy and balanced intestinal microbiome provides human benefits to the host, whereas disruption of the microbiota favors pathogen infections. Enteric pathogens may also compete with the microbiome for nutrients and environmental niches. This dynamic, complicated, and sophisticated interaction force the necessity of rethinking the traditional host–pathogen interaction approach. Investigations covering the three-way gameplay between the host, its microbiota, and the infecting pathogen give a unique opportunity to deeply understand what is really happening during pathogenic infection.

Many of the different experimental models mentioned in this review have enabled us to investigate this complex interaction and ultimately add new data to the field of pathogen infection. To maintain the balance between costs and potential gain, the investigator’s needs and expectations should be precisely determined. It seems reasonable to start with cheap and relatively easy-to-use media flow models, followed by a creative combination of the aforementioned *in vitro* and *in vivo* models. The use of organoids and gut-on-a-chip may push *in vitro* interactions in the microbiota context to a higher complexity level. One step further is the genetically modified cell lines and animals with an enhanced or silenced expression of specific receptors, which may impact both microbiota and pathogen behavior. Another interesting idea is to investigate the molecular details at the single-cell level using high-throughput methods, which allow screening of entire microbial and host populations. Comparison of different models when addressing the experimental question may bring a complete, full set of information, starting with molecular events at the level of the particular cell and ending with the monitoring of animal infection from the perspective of populations. Finally, merging sophisticated bioinformatics methods with the traditional experimental part may shed new light on this host–pathogen–microbiota triangle of interactions.

## Author Contributions

KG conceptualized, wrote the original draft and final version of the manuscript, visualized, and prepared the figures.

## Conflict of Interest

The author declares that the research was conducted in the absence of any commercial or financial relationships that could be construed as a potential conflict of interest.

## Publisher’s Note

All claims expressed in this article are solely those of the authors and do not necessarily represent those of their affiliated organizations, or those of the publisher, the editors and the reviewers. Any product that may be evaluated in this article, or claim that may be made by its manufacturer, is not guaranteed or endorsed by the publisher.
